# Efficacy of lorlatinib after alectinib-induced interstitial lung disease in a patient with anaplastic lymphoma kinase-positive non-small cell lung cancer: a case report

**DOI:** 10.1186/s13256-022-03556-8

**Published:** 2022-08-24

**Authors:** Fumihiro Kashizaki, Arihito Tanaka, Yasutomo Sekido

**Affiliations:** 1 Department of Respiratory Medicine, Yokohama Minami Kyosai Hospital, 1-21-1 Mutsuurahigashi, Kanazawa-ku, Yokohama, Kanagawa 236-0037 Japan; 2 Department of Respiratory Medicine, Isehara Kyodo Hospital, 345 Tanaka, Isehara, Kanagawa 259-1187 Japan; 3 Department of Pathology, Isehara Kyodo Hospital, 345 Tanaka, Isehara, Kanagawa 259-1187 Japan

**Keywords:** Anaplastic lymphoma kinase, Non-small cell lung cancer, Alectinib, Lorlatinib, Drug-induced interstitial lung disease

## Abstract

**Background:**

Anaplastic lymphoma kinase tyrosine kinase inhibitors are standard therapeutic agents prescribed for anaplastic lymphoma kinase-positive non-small cell lung cancer, and treatment with these agents has been shown to contribute to long-term survival in patients. However, there is no consensus regarding the course of treatment after the onset of anaplastic lymphoma kinase tyrosine kinase inhibitors related drug-induced interstitial lung disease.

Here, we present a case of successful lorlatinib treatment after the onset of drug-induced interstitial lung disease caused by alectinib.

**Case presentation:**

A 57-year-old Japanese man was diagnosed with stage IVB non-small cell lung cancer by bronchoscopy, but gene mutation testing could not be performed because of the small amount of specimen. After diagnosis, first-line therapy with cisplatin/pemetrexed was initiated, but the patient developed renal dysfunction. Bronchoscopy was performed again to guide further treatment, and the non-small cell lung cancer was found to be anaplastic lymphoma kinase positive. Alectinib was started after the onset of progressive disease, but it resulted in drug-induced interstitial lung disease, necessitating alternative treatments. He subsequently received nanoparticle albumin bound paclitaxel, which was halted in view of the renal dysfunction. Thereafter, lorlatinib was administered, which was continued without drug-induced interstitial lung disease relapse.

**Conclusion:**

Since alectinib can occasionally cause drug-induced interstitial lung disease, as in the present case, lorlatinib may be an option to continue treatment in patients without other treatment alternatives.

## Background

With the identification of gene abnormalities in anaplastic lymphoma kinase (ALK)-positive non-small cell lung cancer (NSCLC), effective ALK tyrosine kinase inhibitors (ALK-TKIs) have been developed for treatment. ALK-TKIs are more effective than cytotoxic chemotherapy, and long-term treatment with TKIs has been shown to prolong the overall survival of patients. Lorlatinib is a novel third-generation ALK TKI that has been shown to be more potent than second-generation TKIs in biochemical and cellular assays and has the broadest coverage of the identified ALK resistance mutations. Additionally, it was designed to cross the blood–brain barrier so as to achieve high exposure levels in the central nervous system (CNS) [1]. Because of its efficacy, lorlatinib is the standard treatment option for patients with ALK-positive NSCLC for whom one or more ALK-TKIs have failed.

However, it is unclear whether lorlatinib can be used after discontinuation of another ALK-TKI owing to adverse events, for example, in cases of drug-induced interstitial lung disease (DILD). Here, we present a rare case showing the feasibility of continuing lorlatinib after the onset of DILD caused by alectinib.

## Case presentation

**Fig. 1:**
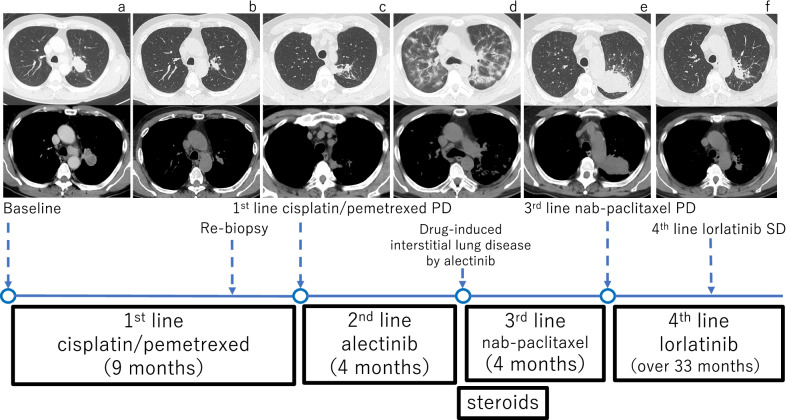
Treatment course. First-line treatment with cisplatin/pemetrexed shrunk the primary lesion and mediastinal lymph nodes (**a**, **b**), but PD was noticed 9 months after the beginning of treatment (**c**). Re-biopsy performed during treatment with cisplatin/pemetrexed indicated that the tumor was ALK-positive. Therefore, second-line treatment with alectinib was subsequently initiated. Although alectinib treatment was successful, the patient developed DILD 4 months after the start of treatment (**d**). After DILD was relieved upon treatment with steroids, third-line treatment with nab-paclitaxel was initiated, but PD was observed 4 months later (**e**). Thereafter, fourth-line treatment with lorlatinib was started and has been continued for more than 33 months while maintaining SD (**f**). *PD* progressive disease, *ALK* anaplastic lymphoma kinase, *DILD* drug-induced interstitial lung disease, *SD* stable disease, *nab-paclitaxel* nanoparticle albumin bound paclitaxel

**Fig. 2.**
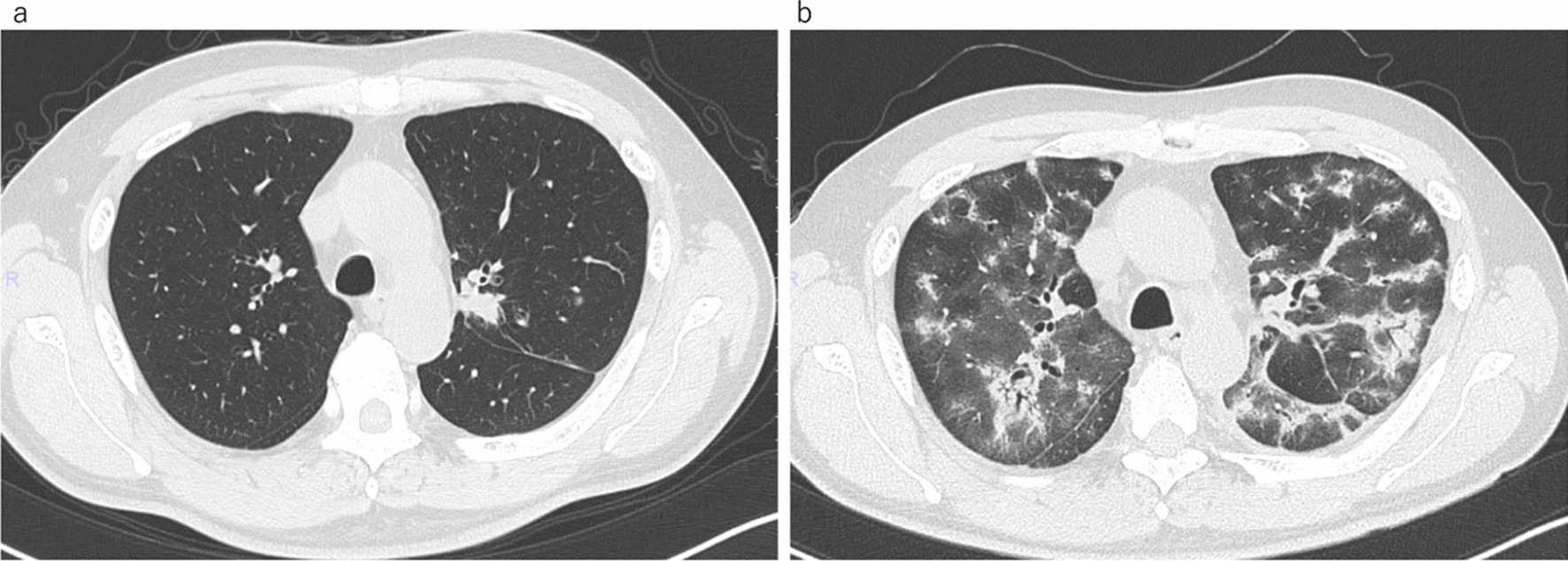
Thin-slice CT before and after the onset of DILD. Prior to the onset of DILD (**a**), a shadow of the primary lesion was observed in the left upper lobe of the lung. No abnormal shadow was seen in the lung field. After the patient developed DILD (**b**), the primary lesion was observed to be shrinking. However, ground-glass opacities and infiltrations were seen spreading on both sides. *CT* computed tomography, *DILD* drug-induced interstitial lung disease

**Fig. 3:**
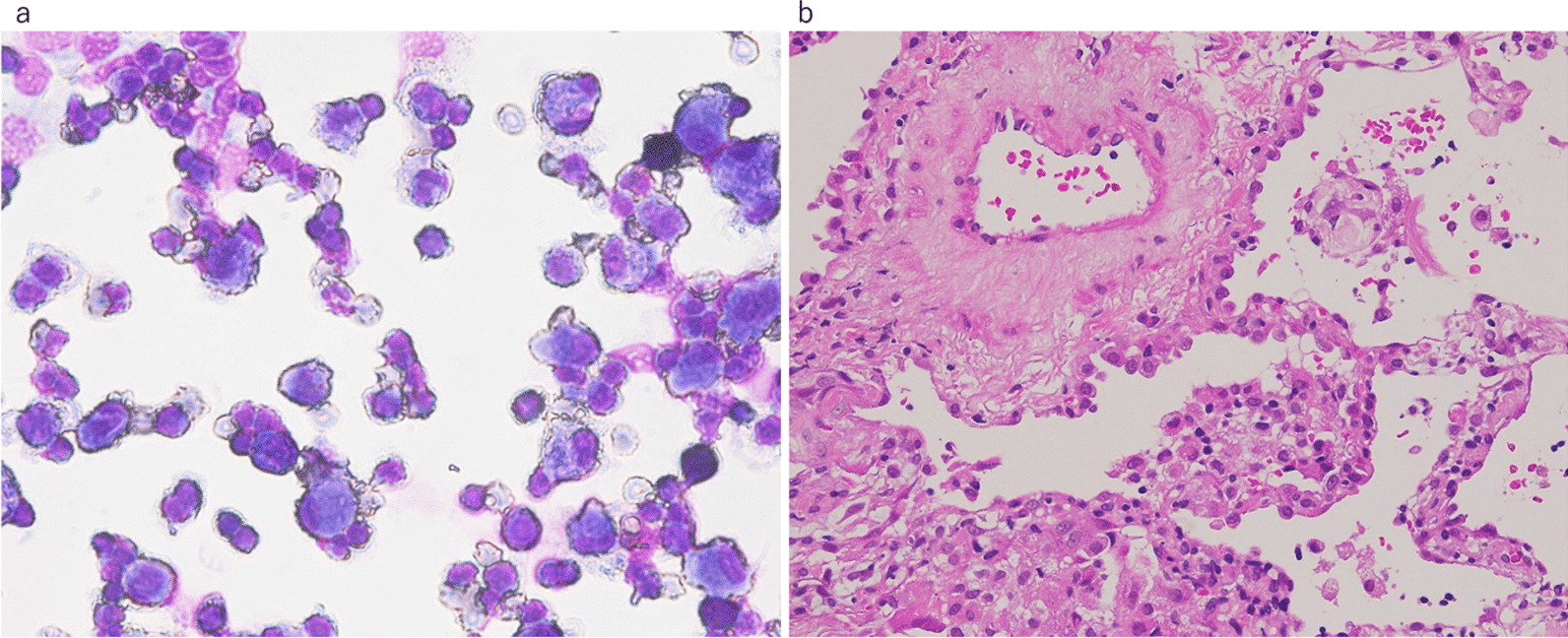
BALF and TBLB at the onset of DILD. Giemsa staining of the BALF at a magnification of ×200 (**a**) showing an increase in the proportion of lymphocytes (34.4%). Hematoxylin and eosin staining of the TBLB specimen at a magnification of ×200 (**b**) showing exudate and inflammatory cell infiltration in the alveolar space with alveolar wall thickness. No findings suggestive of cancer infiltration were observed. *BALF* bronchoalveolar lavage fluid, *TBLB* transbronchial lung biopsy, *DILD* drug-induced interstitial lung disease

**Fig. 4:**
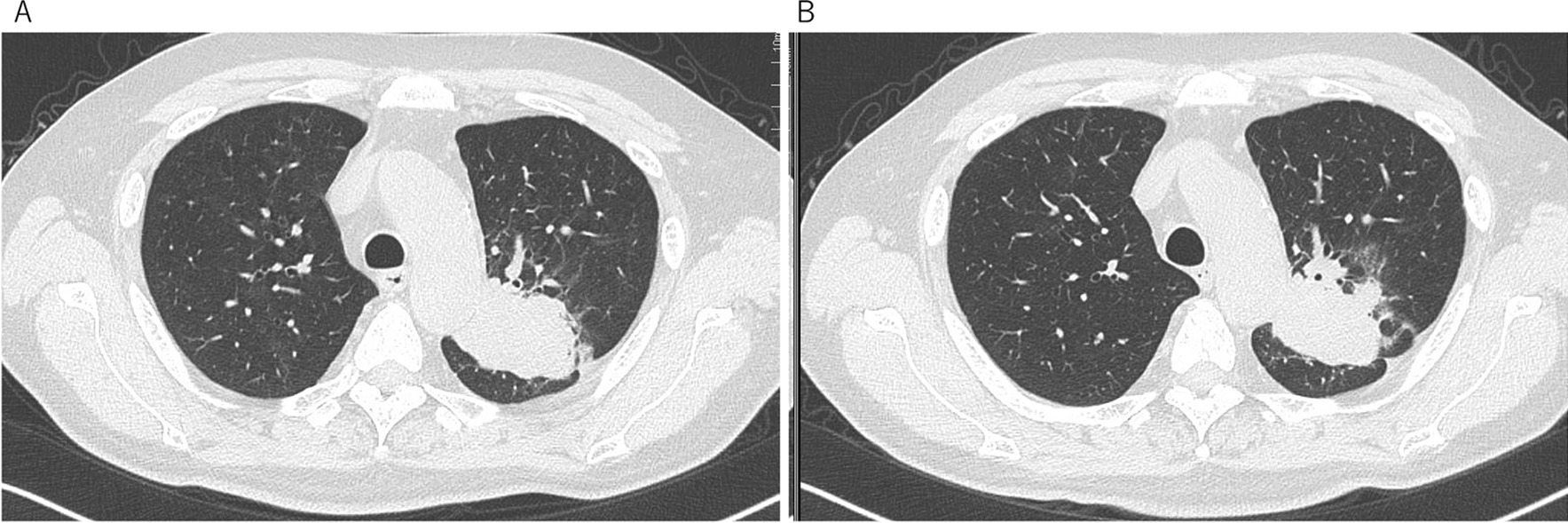
Thin-slice CT before and after the administration of lorlatinib. Compared with the patient’s condition before the administration of lorlatinib (**a**), SD has been maintained 33 months after the start of lorlatinib (**b**), and no recurrence of DILD has been observed. *CT* computed tomography, *SD* stable disease, *DILD* drug-induced interstitial lung disease

A 57-year-old Japanese man with a chief complaint of hoarseness visited the Otolaryngology Department at our hospital. A contrast-enhanced computed tomography (CT) scan showed a tumor, with its major axis measuring approximately 40 mm in the left upper lobe of the lung. The hilar and mediastinal lymph nodes were also swollen (Fig. 1a). Primary lung cancer was suspected, and the patient was directed to the Department of Respiratory Medicine on the same day that he was admitted to the Otolaryngology Department. He was diagnosed with lung adenocarcinoma, which was confirmed by bronchoscopy. We performed transbronchial lung biopsy (TBLB) for the primary lesion in the left upper lobe and endobronchial ultrasonography-guided transbronchial needle aspiration in the mediastinal lymph nodes 1 week after the first visit. Genetic mutation testing was not performed because the sample was insufficient. Subsequently, ^18^F-fluorodeoxyglucose positron emission tomography/CT (^18^F-FDG PET/CT) was performed, and abnormal accumulation was observed in the longitudinal hilar lymph nodes (#4L, #5, #10L), vertebral body, and left pleura. The results of the ^18^F-FDG PET/CT and other findings led to the diagnosis of lung adenocarcinoma [cT2bN2M1c (PUL, OSS); stage IVB]. We recommended rebiopsy considering the possibility of a genetic mutation, but the patient refused the examination because of strong coughing during the first bronchoscopy and progressive symptoms of hoarseness. The patient strongly desired an early treatment, so we initiated first-line therapy with cisplatin (75 mg/m^2^) and pemetrexed (500 mg/m^2^) 3 weeks after the first visit. However, the patient developed grade 2 renal dysfunction [creatinine, 1.83 mg/dL; estimated glomerular filtration rate (eGFR), 31.23 mL/min/1.73 m^2^], making it difficult to continue the therapy (Fig. 1b). We informed the patient about the need for a second bronchoscopy for consideration of further treatment and obtained his consent. Bronchoscopy was performed again, and the patient was diagnosed with ALK-positive NSCLC (immunohistochemistry, 3+; fluorescence *in situ* hybridization, 56%). In addition, progressive disease was noted (Fig. 1c). Development of progressive disease required approximately 9 months, and cisplatin and pemetrexed were administered over four cycles, after which pemetrexed alone was administered over five cycles. Thereafter, second-line therapy with alectinib (300 mg/day) was initiated. Three months after starting alectinib, chest CT showed that the primary lesion had shrunk, and we judged it to be a partial response, but the ground-glass opacities and infiltration continued to spread on both sides (Figs. 1d, 2). At this time, the patient complained of dyspnea on exertion, and his saturation of percutaneous oxygen was approximately 82% in room air. Blood gas analysis showed that the partial pressure of arterial oxygen was 58.2 torr, indicating respiratory failure. A pulmonary function test revealed restrictive ventilatory impairment, with a vital capacity (VC) of 2.11 L and a %VC of 57.1%. A diffuse lung capacity for carbon monoxide (DLCO) of 4.84 mL/min/mmHg and a %DLCO of 22.8% indicated diffusion disorder. Bronchoalveolar lavage fluid (BALF) (Fig. 3a) and TBLB samples (Fig. 3b) were obtained at this time. The BALF showed an increased lymphocyte ratio (34.4%), and pathological analysis of the TBLB specimen revealed an organizing pneumonia (OP)/nonspecific interstitial pneumonia (NSIP) overlapping pattern. On the basis of the chest CT results, we suspected alectinib-induced DILD. However, we also considered respiratory infection or cancerous lymphangiopathy with cancer progression in the differential diagnoses. Laboratory testing revealed a white blood cell count of 9300/µL and a C-reactive protein level of 1.41 mg/dL. The BALF culture results were negative. The BALF was lymphocyte dominant, and no findings suggestive of cancer infiltration were observed in the TBLB specimen. On the basis of these results, we excluded the other conditions evaluated in the differential diagnosis. Our final diagnosis was alectinib-induced DILD. On the basis of the results of chest CT and bronchoscopic pathology, methylprednisolone was prescribed (1 g/day for 3 days). The patient then received oral prednisolone (0.8 mg/kg/day), which was gradually reduced, and he was administered steroids for about 3 months. Third-line therapy with nanoparticle albumin bound paclitaxel (nab-paclitaxel)  (100 mg/m^2^) was initiated after treatment with steroids. However, 3 months later, obstructive pneumonia associated with an increase in the size of the primary lesion was observed (Fig. 1e). Moreover, mild renal dysfunction persisted (creatinine, 1.63 mg/dL; eGFR, 35.45 mL/min/1.73 m^2^). Because of this complication and upon the patient’s request for oral medication, lorlatinib (100 mg/day) was administered as fourth-line therapy. Treatment with antibiotics and lorlatinib reduced the lesion’s shadow, and no recurrence of DILD was observed. Thirty-three months after the start of treatment, stable disease has been maintained, and lorlatinib has been continued (Figs. 1f, 4).

## Discussion and conclusions

In the search for advanced lung cancer treatment, driver genes have been identified, and several molecular-targeted drugs have been developed and reported to exhibit remarkable antitumor effects. For ALK-positive NSCLC, alectinib was more effective than crizotinib in phase III trials (ALEX study) and is, therefore, widely used for first-line treatment [2]. Lorlatinib and ceritinib are effective against alectinib-resistant lung cancer and have been used in second-line treatments, based on the status of the resistance gene [3]. Lorlatinib, which is derived from crizotinib, is structurally optimized to inhibit mutations conferring resistance, including G1202R, and those with a high rate of migration to the CNS [3]. In the CROWN trial, the intracranial response among patients with measurable brain metastases at baseline was 82%, with a complete intracranial response rate of 71%. In the global ALEX, ALTA-1L, and eXalt3 trials, the corresponding complete intracranial response rates with alectinib, brigatinib, and ensartinib were 38%, 28%, and 27%, respectively [1, 4]. Moreover, in the CROWN trial, lorlatinib significantly decreased the cumulative incidence of CNS progression, which suggests that the prolonged progression-free survival seen with lorlatinib may be partly due to the prevention of CNS metastases [1]. In the CROWN trial, progression-free survival was significantly longer among patients with ALK-positive NSCLC who received first-line lorlatinib than among those who received crizotinib. In untreated patients, lorlatinib may eliminate rare preexisting subclones harboring ALK resistance mutations or prevent the emergence of such resistant subclones [1].

However, a molecular-targeted drug with a clear antitumor effect may still have to be discontinued upon the occurrence of side effects. The overall frequency of DILD associated with ALK-TKIs is 2.14% [5]. Although the incidence of DILD secondary to ALK-targeted therapy is low, it carries a 50% risk of mortality according to a previous case series [6]. Moreover, there is no consensus regarding the optimal treatment approach for ALK-TKI-induced DILD or continued treatment for NSCLC after remission of DILD. A limited number of previous case reports have described the successful administration of crizotinib, ceritinib, alectinib, and brigatinib in patients with ALK-rearranged NSCLC who have recovered from DILD secondary to ALK-targeted therapy [7]. A report of two cases showed that treatment with lorlatinib was successful after treatment with steroids for DILD caused by alectinib [7]. However, bronchoscopy, including BALF and TBLB, was not performed in these two patients because their respiratory condition was poor. Therefore, this is the first case in which lorlatinib was administered after treatment with steroids for DILD caused by alectinib after other diseases, such as cancerous lymphangiopathy and infectious diseases, could be ruled out by BALF and TBLB to some extent. In this case, alectinib-induced DILD showed an organizing pneumonia with nonspecific interstitial pneumonia overlapping pattern (OP/NSIP) on chest imaging findings, the BALF was lymphocyte dominant, and the TBLB findings were also consistent with OP/NSIP. Therefore, we expected that steroids would be effective, and when we administered steroids, the DILD improved and we were able to continue treatment for NSCLC.

As of August 2022, no previous study has reported serious lung disease induced by lorlatinib, and it can be administered in normal doses even to patients with renal dysfunction [8]. As seen in the present case, alectinib can sometimes cause DILD. Therefore, lorlatinib is a viable alternative. Despite their side effects, ALK-TKIs prolong overall survival. The use of an alternative ALK-TKI with proper management of side effects may extend the positive effects on overall survival. Evidence from future studies will help determine the optimal chemotherapy regimen after the onset of ALK-TKI-associated DILD.

In summary, in patients with driver mutation-positive NSCLC, continuation of treatment with TKIs to the maximum extent possible is essential to improve the prognosis. Even after TKI-induced DILD, another TKI may be considered as a treatment option.

## Data Availability

The dataset used and analyzed during the current study is available from the corresponding author on reasonable request.

## Abbreviations

ALK: Anaplastic lymphoma kinase
TKIs: Tyrosine kinase inhibitors
NSCLC: Non-small cell lung cancer
DILD: Drug-induced interstitial lung disease
CNS: Central nervous system
CT: Computed tomography
TBLB: Transbronchial lung biopsy
^18^F-FDG PET/CT: ^18^F-fluorodeoxyglucose positron emission tomography/computed tomography
VC: Vital capacity
DLCO: Diffuse lung capacity for carbon monoxide
BALF: Bronchoalveolar lavage fluid
OP: Organizing pneumonia
NSIP: Nonspecific interstitial pneumonia
